# The Effects of Malaria in Pregnancy on Neurocognitive Development in Children at 1 and 6 Years of Age in Benin: A Prospective Mother–Child Cohort

**DOI:** 10.1093/cid/ciab569

**Published:** 2021-07-23

**Authors:** Amanda Garrison, Michael J Boivin, Nadine Fiévet, Roméo Zoumenou, Jules M Alao, Achille Massougbodji, Michel Cot, Florence Bodeau-Livinec

**Affiliations:** 1 Ecole des Hautes Etudes en Santé Publique (EHESP), Rennes, France; 2 Université de Paris, Center of Research in Epidemiology and Statistics (CRESS), Institut National de la Santé et de la Recherche Médicale, Institut National de la Recherche Agronomique, Paris, France; 3 Sorbonne Universités, Université de Paris, Paris, France; 4 Departments of Psychiatry and Neurology/Ophthalmology, Michigan State University, East Lansing, Michigan, USA; 5 Université de Paris, Mère et Enfant Face aux Infections Tropicales, Institut de Recherche Pour le Développement, Paris, France; 6 Service de Pédiatrie, Centre Hospitalier Universitaire de la Mère et de l’Enfant–Lagune de Cotonou, Cotonou, Benin; 7 Faculté des Sciences de la Santé, Université d’Abomey-Calavi, Cotonou, Benin

**Keywords:** child development, malaria, neurocognition, pregnancy, sub-Saharan Africa

## Abstract

**Background:**

Malaria in pregnancy (MiP) contributes significantly to infant mortality rates in sub-Saharan Africa and has consequences on survivors, such as preterm birth and low birth weight. However, its impact on long-term neurocognitive development in children remains unknown.

**Methods:**

Our prospective cohort included pregnant women and their live-born singletons from the Malaria in Pregnancy Preventive Alternative Drugs clinical trial. MiP was assessed using microscopy and real-time quantitative polymerase chain reaction (qPCR). Neurocognitive development in children was assessed using the Mullen Scales of Early Learning and the Kaufman Assessment Battery for Children, 2nd edition (KABC-II), at 1 and 6 years of age, respectively.

**Results:**

Of 493 pregnant women, 196 (40%) were infected with malaria at least once: 121 (31%) with placental malaria diagnosed by qPCR. Multiple linear regression B-coefficients showed that impaired gross motor scores were associated with MiP at least once (−2.55; confidence interval [95% CI]: −5.15, 0.05), placental malaria by qPCR (−4.95; 95% CI: −7.65, −2.24), and high parasite density at delivery (−1.92; 95% CI: −3.86, 0.02) after adjustment. Malaria and high parasite density at the second antenatal care visit were associated with lower KABC-II Non-Verbal Index scores at 6 years (−2.57 [95% CI: −4.86, −0.28] and −1.91 [−3.51, −0.32]), respectively.

**Conclusions:**

This prospective cohort study provides evidence that MiP, particularly late term, could have important negative consequences on child development at 1 and 6 years of age. Mechanisms behind this association must be further investigated and diagnostic methods in low-income countries should be strengthened to provide adequate treatment.

**Clinical Trials Registration:**

NCT00811421.

## Teaser

This paper provides the first evidence of an association between late-term peripheral and placental malaria in pregnancy and impaired neurocognitive development in offspring at 1 and 6 years of age in Benin, sub-Saharan Africa.

Malaria remains one of the greatest contributors to morbidity and mortality in areas with stable transmission, particularly in sub-Saharan Africa (SSA). Of the 25 million pregnant women in SSA countries at risk of malaria infection, an estimated 25% will have evidence of *Plasmodium falciparum* placental malaria at the time of delivery [1] and 50% will have evidence of peripheral malaria in the blood during antenatal care follow-up [2]. This high prevalence is due to the fact that pregnant women are at increased risk of contracting malaria compared with nonpregnant women of the same age [1].

While malaria in pregnancy (MiP) contributes to approximately 10 000 maternal and 200 000 fetal deaths [3], it can also have significant consequences on survivors. Malaria in pregnancy is associated with increased risk of maternal anemia, fetal growth restriction, low birth weight, preterm birth, and later malaria infection in offspring [4, 5]. Laboratory simulations have demonstrated that experimental malaria-exposed offspring also had persistent neurological deficits compared with the control group [6]. Researchers hypothesized that the activation of the maternal inflammatory immune response may impact fetal brain development in the placenta, therefore resulting in neurodevelopmental impairments [7]. To date, the direct link between MiP and neurocognitive impairment in children has yet to be investigated prospectively in SSA populations. Therefore, the main objective of this paper was to assess the impacts of MiP, including peripheral and placental malaria, on neurocognitive and motor development scores in surviving children at 1 and 6 years of age in a prospective mother–child cohort in Benin.

## METHODS

### Ethics Statement

Ethical approval for all studies mentioned in this article was obtained by the institutional review boards of the Beninese Ethical Committee of the Faculté des Sciences de la Santé (FSS) and the Committee of Ethical Research of the Applied Biomedical Sciences Institute (CER-ISBA) in Benin, New York University in the United States (IRB #09-1253), and the Research Institute for Development’s (IRD) Consultative Ethics Committee in France. Written informed consent was obtained or thumbprints were provided for consent if women could not read or write.

### Study Population

Pregnant women in this prospective mother–child cohort were enrolled in the Malaria in Pregnancy Preventive Alternative Drugs (MiPPAD) clinical trial (NCT00811421) during their second trimester. The objective of the trial was to compare the efficacy of 2 intermittent preventive treatments of MiP (IPTp), sulfadoxine-pyrimethamine (SP) and mefloquine (MQ), in 3 health centers in the Allada district in Benin (Sékou, Allada, and Attogon). Information on inclusion and exclusion criteria is detailed in a preceding publication [8]. Briefly, inclusion criteria for women were as follows: a permanent residence in the study area, gestational age of 28 weeks or less, human immunodeficiency virus (HIV) negative at recruitment, no known allergies to sulfa or mefloquine drugs, no treatment of malaria using these drugs within 4 weeks of recruitment, and no history of severe renal, hepatic, psychiatric, or neurological disease. Participants were recruited into a nested-cohort study, Anemia in Pregnancy: Etiologies and Consequences (APEC), to be followed up for parasitic infection during pregnancy. All eligible surviving singletons were invited to undergo neurocognitive assessments in the TOVI (“child” in *Fon*) study 1 year after birth [9]. Surviving children were followed up at 6 years of age for further neurocognitive assessments in the EXPLORE (EXposition au PLomb et au manganèse et Risques pour l’Enfant) study.

### Malaria in Pregnancy

As part of the Benin pregnancy package, women were given IPTp (1500/75 mg SP per dose or 15 mg/kg MQ per dose) and iron and folic acid supplements during 3 antenatal care visits (ANVs) within the clinical trial, and mebendazole to treat helminth infection if present. Clinical malaria episodes were treated with oral quinine in the first trimester or with artemether-lumefantrine in subsequent trimesters for uncomplicated malaria; severe malaria was treated with parental quinine [10]. Malaria was detected using thick blood smears that were stained and read according to standard quality-controlled procedures during pregnancy [11]. At delivery, maternal peripheral and placental blood samples were collected. Blood samples were stained on filter paper and tested for the presence and density of *P. falciparum* using real-time quantitative polymerase chain reaction (qPCR) tests targeting 18S rDNA. Parasitemia was quantified by extrapolation of cycle thresholds, the value for which DNA is amplified, from a standard curve of *P. falciparum* ring-infected erythrocytes. Samples where no cycle thresholds were detected were considered negative, and a density of 2 parasites/μL was assigned for positive samples if amplification was lower than the standard curve of 5 parasites/μL [12].

### Child Neurocognitive Development

At 1 year of age, trained research nurses assessed cognitive function in infants using the Mullen Scales of Early Learning (MSEL) from April 2011 until November 2012. The MSEL is composed of 5 scales for gross motor, fine motor, visual reception, receptive language, and expressive language. Quality assurance of assessments, including interrater reliability, was established and the MSEL was adapted for use in this population in a pilot study [13]. Normalized age-specific t-scores of the visual reception, fine motor, receptive language, and expressive language scales were combined to form the Early Learning Composite (ELC) score to indicate overall early cognitive function. The ELC and gross motor scores were analyzed in this paper. The Home Observation for Measurement of the Environment (HOME) inventory, adapted for use in this setting, assessed the quality of the home environment and parent–child interactions at 1 year.

Neurocognitive development in children at 6 years was assessed using the Kaufman Assessment Battery for Children, 2nd edition (KABC-II), a psychological diagnostic tool that assesses processing ability, cognitive ability, and planning and learning capabilities. The French version of the KABC-II was orally translated into the local language by trained investigators and the construct validity for use in this population was verified during a pilot study [14]. The Mental Processing Index (MPI) and the Non-Verbal Index were used in analyses. The Bruininks-Oseretsky Test of Motor Proficiency, 2nd edition (BOT-2), was administered to evaluate fine and gross motor skills in children. A final, composite score for fine and gross motor skills was standardized by age and sex and used in analyses.

### Sociodemographic Characteristics

At recruitment, information on gravidity, iron deficiency, IPTp treatment, and sociodemographic characteristics such as maternal age, education, and socioeconomic status was collected. Soil-transmitted helminth (STH) infection was diagnosed in stool samples of women using the Kato-Katz technique. Prepregnancy body mass index (BMI) was calculated using the gestational age at inclusion, according to fundal height using manual palpation and estimated expected weight gain of women during pregnancy. At delivery, information on infant sex, gestational age according to the Ballard score, and birth weight in grams was collected. Risk factors for MiP and impaired child neurocognitive development were identified using direct acyclic graphs.

### Statistical Analysis

Statistical analysis involved the description of sociodemographic and clinical characteristics of women during pregnancy and infant birth outcomes. Characteristics are shown for mother–child pairs included in analyses, those lost to follow-up, and deceased children. Univariate analyses and Kruskal-Wallis, Wilcoxon rank-sum, and chi-square tests were used to test the significance of common risk factors for exposures and outcomes. Linear regression models with bootstrapped 95% confidence intervals (95% CIs) tested associations between malaria diagnosed during pregnancy by microscopy and qPCR and neurocognitive development outcomes in children at 1 and 6 years. Sensitivity analyses were conducted to ensure the robustness of results, including the multiple imputation of missing variables. Analyses were conducted using STATA 16 (StataCorp 2019, Stata Statistical Software: release 16; StataCorp LLC, College Station, TX). Statistical tests were 2-tailed with an ɑ risk of 5%.

## RESULTS

**Figure 1.**
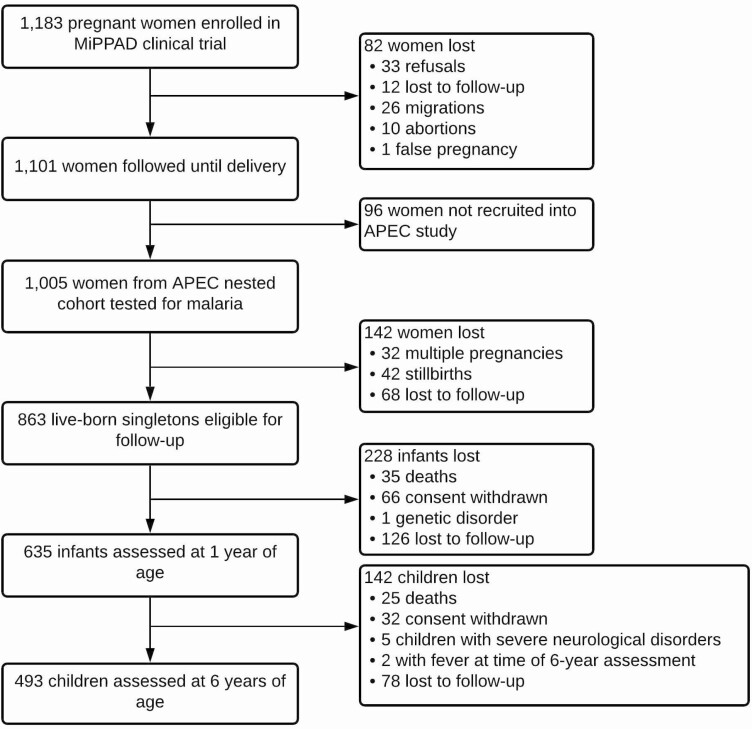
Population follow-up of women and children from pregnancy until 6 years of age. Abbreviations: APEC, Anemia in Pregnancy: Etiologies and Consequences; MiPPAD, Malaria in Pregnancy Preventive Alternative Drugs.

Of the 1183 pregnant women recruited into the MiPPAD clinical trial, the first 1005 women were asked to participate in the APEC nested-cohort study (Figure 1). At delivery, 863 eligible singletons were born alive and 635 were assessed for neurocognitive development at 1 year. Finally, 493 were further assessed for neurocognitive development at 6 years.

### Description of Population

Sociodemographic and clinical characteristics of mother–child pairs in our analyses during pregnancy and at birth are described and compared with those lost to follow-up and deceased before 6 years of age in Table 1. The prevalence of peripheral MiP was 15% at the first ANV, 3% at the second ANV, and 10% at delivery. At delivery, 31% of women tested had placental malaria according to qPCR analyses. There were no significant differences in malaria infection or parasite density between those included in analyses and lost to follow-up. Children who died between birth and 6 years of age were more likely to have been born primigravida, been preterm (<37 weeks’ gestation), and have low birth weight (<2500 g). There was no difference in MiP exposure between children followed at 6 years and those deceased.

**Table 1.** Comparison of Maternal and Birth Characteristics During Pregnancy in Mother–Child Pairs Included in Analyses, Lost to Follow-up, and Children Deceased Between Birth and 6 Years of Age

Parameter	Missing (n/493), %	Included (n = 493), n (%)	Lost to Follow-up (n = 310), n (%)	*P*	Deceased (n = 60), n (%)	*P*
Age at delivery	0			.10		.47
* <*25 y		255 (52)	179 (58)		34 (57)	
* *>25 y		238 (48)	131 (42)		26 (43)	
Gravidity	0			.04		.05
* *Primigravida		75 (15)	65 (21)		15 (25)	
* *Multigravida		418 (85)	245 (79)		45 (75)	
Prepregnancy BMI (kg/m^2^)	2 (<1)			.12		.50
* *Underweight		49 (10)	43 (14)		3 (5)	
* *Normal		353 (72)	219 (72)		44 (76)	
* *Overweight/obese		89 (18)	44 (14)		11 (19)	
IPTp treatment	0			.31		.38
* *SP		161 (33)	112 (36)		23 (38)	
* *MQ		332 (67)	198 (64)		37 (62)	
Iron deficiency^a^	0			.08		.12
* *No		173 (35)	90 (29)		15 (25)	
* *Yes		320 (65)	220 (71)		45 (75)	
STH infection^b^	2 (<1)			.12		.70
* *No		396 (81)	260 (85)		48 (83)	
* *Yes		95 (19)	46 (15)		10 (17)	
Maternal education	0			.04		.06
* *None		337 (68)	190 (61)		48 (80)	
* *Primary+		156 (32)	120 (39)		12 (20)	
Family possession score (quartile)^c^	0			.004		.02
* *1		147 (30)	122 (39)		15 (25)	
* *2		105 (21)	74 (24)		15 (25)	
* *3		201 (41)	89 (29)		18 (30)	
* *4		40 (8)	25 (8)		12 (20)	
Preterm (<37 weeks’ gestation)	10 (2)			.75		.01
* *No		450 (93)	274 (93)		48 (83)	
* *Yes		33 (7)	22 (7)		10 (17)	
Low birth weight (<2500 g)	38 (8)			.46		<.001
* *No		417 (92)	253 (90)		39 (71)	
* *Yes		38 (8)	28 (10)		16 (29)	
Child sex	0			.27		.26
* *Male		252 (51)	146 (47)		26 (43)	
* *Female		241 (49)	164 (53)		34 (57)	
Thick blood smear at first ANV	0			.61		.97
* *Negative		420 (85)	260 (84)		51 (85)	
* *Positive		73 (15)	50 (16)		9 (15)	
Thick blood smear at second ANV	6 (1)			.41		.48
* *Negative		470 (97)	285 (95)		53 (95)	
* *Positive		17 (3)	14 (5)		3 (5)	
Thick blood smear at delivery	26 (5)			.70		.41
* *Negative		418 (90)	245 (90)		53 (93)	
* *Positive		49 (10)	26 (10)		4 (7)	
qPCR of placenta	100 (20)			.69		.93
* *Negative		271 (69)	136 (67)		32 (70)	
* *Positive		121 (31)	66 (33)		14 (30)	
MiP at least once in pregnancy	0			.57		.48
* *No		297 (60)	193 (62)		39 (65)	
* *Yes		196 (40)	117 (38)		21 (35)	
Parasite density at first ANV^d^	0	587 (9–35 940)	570(12–100 391)	.95	1301 (12–8291)	.45
Parasite density at second ANV^d^	0	231 (3–63 090)	1128 (3–19 700)	.93	24 (3–11 230)	.29
Parasite density at delivery^d^	0	84 (0.5–859 446)	165 (2–1 185 727)	.98	798 (2–352 534)	.55

Abbreviations: ANV, antenatal visit; BMI, body mass index; IPTp, intermittent preventive treatments of MiP; MiP, malaria in pregnancy; MQ, mefloquine; qPCR, quantitative polymerase chain reaction; SP, sulfadoxine-pyrimethamine; STH, soil-transmitted helminth.

^a^Iron deficiency at recruitment, defined as ferritin serum concentration < 12 μg/L in the absence of inflammation or a serum ferritin concentration <70 μg/L in the presence of inflammation measured by C-reactive protein.

^b^Women considered to be infected with STHs if the fecal egg count (FEC) was at least 24 eggs per gram for any species.

^c^Family possession score was computed using a checklist of material possessions that families could own and indicated family wealth: radio, television, bicycle, motorbike, car, at least 2 cows, and electricity (quartile 1 = most deprived, quartile 4 = least deprived).

^d^Parasite density is shown in parasites/μL; parasite density at delivery was determined through qPCR. The median (range) of parasite densities is shown.

### Univariate Analyses

Factors associated with MiP and cognitive development identified in the literature are shown in Tables 2 and 3. Prepregnancy BMI, STH infection during pregnancy, maternal education, family possession score, the HOME score, low birth weight, and malaria in childhood were found to be significantly associated with both cognitive functioning and gross motor scores in children at 1 year. Maternal age, prepregnancy BMI, maternal education, family possession score, the HOME score, and child sex were associated with cognitive and motor development in children at 6 years. In addition, gravidity was associated with motor development in children at 1 and 6 years.

**Table 2.** Associations Between Risk Factors and Neurocognitive Development Measured by the MSEL Early Learning Composite (ELC) and the Gross Motor Composite Score at 1 Year

Parameters	Neurocognitive Assessments at 1 Year			
	MSEL ELC	*P*	Motor Composite	*P*
Maternal characteristics				
* *Age at delivery		.68		.16
* <*25 y	100 (14)		48.5 (25)	
* *>25 y	100 (20)		49 (20)	
* *Gravidity		.34		.04
* *Primigravida	102 (15)		45.5 (20)	
* *Multigravida	99 (18)		49 (19)	
* *Prepregnancy BMI (kg/m^2^)		.10		.06
* *Underweight	102 (13)		55 (18)	
* *Normal	99 (16)		49 (19)	
* *Overweight/obese	98 (20)		44 (19)	
* *IPTp treatment		.20		.29
* *SP	101 (18)		49 (23)	
* *MQ	99 (18)		49 (19)	
* *Iron deficiency^a^		.76		.81
* *No	100 (17)		49 (19)	
* *Yes	100 (18)		49 (19)	
* *STH infection^b^		.002		.03
* *No	101 (15)		49 (20)	
* *Yes	94.5 (23.5)		48 (21)	
* *Maternal education		<.001		<.001
* *None	97 (18)		48 (24)	
* *Primary+	104 (13)		49 (23)	
* *Family possession score (quartile)^c^		.001		.01
* *1	97 (19)		44.5 (24)	
* *2	95 (20)		48 (15)	
* *3	102 (15)		49 (23)	
* *4	103 (13.5)		49 (17)	
* *EPDS score^d^		.31		.75
* <*10	99 (18)		49 (19)	
* *>10	101 (18)		49 (19)	
* *HOME score^e^		<.001		.002
* <*27 (mean)	98 (20)		47.5 (25)	
* *>27	102 (14)		49 (20)	
Child characteristics				
* *Preterm (<37 weeks’ gestation)		.02		.31
* *No	101 (15)		49 (19.5)	
* *Yes	93.5 (26)		48 (20)	
* *Low birth weight (<2500 g)		.06		.008
* *No	100 (16)		49 (20)	
* *Yes	94.5 (18)		43.5 (19)	
* *Malaria infection^f^		.02		.02
* *No	100 (18)		49 (20)	
* *Yes	97 (21)		44 (17)	
* *Sex		.11		.25
* *Male	99 (16)		49 (20)	
* *Female	101 (20)		48 (19)	
* *Blood lead levels^g^		.17		.44
* <*50 μg	101 (16)		49 (19)	
* *>50 μg	96.5 (21)		49 (23)	

Median (interquartile range) assessment scores are shown for each category. Wilcoxon rank-sum and Kruskal-Wallis analyses were used for binary and ordinal variables, respectively. Abbreviations: BMI, body mass index; EPDS, Edinburg Postnatal Depression Scale; HOME, Home Observation for Measurement of the Environment; IPTp, intermittent preventive treatments of MiP; MiP, malaria in pregnancy; MQ, mefloquine; MSEL, Mullen Scales of Early Learning; SP, sulfadoxine-pyrimethamine; STH, soil-transmitted helminth.

^a^Iron deficiency at recruitment, defined as ferritin serum concentration <12 μg/L in the absence of inflammation or a serum ferritin concentration <70 μg/L in the presence of inflammation measured by C-reactive protein.

^b^Women were considered to be infected with STHs if the fecal egg count (FEC) was at least 24 eggs per gram for any species.

^c^Family possession score was computed using a checklist of material possessions that families could own and indicated family wealth: radio, television, bicycle, motorbike, car, at least 2 cows, and electricity (quartile 1 = most deprived, quartile 4 = least deprived).

^d^Women with a score of >10 were considered to have symptoms of postpartum depression.

^e^The HOME score is a continuous score based on a combination of interview questions and observations of parent–child interactions in the home and consists of several subscales, with higher scores indicating better-quality parent–child interactions and home learning environments.

^f^Malaria infection at the time of neurocognitive assessment, diagnosed by thick blood smear.

^g^Blood lead levels measured at the time of neurocognitive assessment using inductively coupled plasma mass spectrometry (ICP-MS; Perkin Elmer Sciex Elan DRC II ICP-MS instrument). Cutoff determined by the Centers for Disease Control and Prevention (CDC) intervention threshold for lead poisoning.

**Table 3.** Associations Between Risk Factors and Neurocognitive Development Measured by the KABC-II, MPI and NVI, and BOT-2 Standard Score at 6 Years

Parameters	Neurocognitive Assessments at 6 Years					
	KABC-II MPI	*P*	KABC-II NVI	*P*	BOT-2	*P*
Maternal characteristics						
* *Age at delivery		.14		.74		.05
* <*25 y	59 (18)		51 (10)		37 (11)	
* *>25 y	59 (17)		51 (10)		39 (13)	
* *Gravidity		.84		.38		.07
* *Primigravida	59 (19)		49 (11.5)		36 (11)	
* *Multigravida	59 (16)		51 (9)		38 (11)	
* *Prepregnancy BMI (kg/m^2^)		.04		.78		.04
* *Underweight	60 (17)		52 (11)		40 (12)	
* *Normal	59.5 (17)		51 (9)		38 (11)	
* *Overweight/obese	57 (16.5)		51 (9)		36.5 (11.5)	
IPTp treatment		.15		.02		.12
* * SP	58 (17)		50.5 (11)		37 (11.5)	
* * MQ	59 (18)		52 (8)		38 (11)	
Iron deficiency^a^		.16		.70		.31
* * ** **No	60 (15)		51 (8)		39 (11)	
* * ** **Yes	58 (17)		51 (11)		37 (12)	
STH infection^b^		.32		.55		.42
* * No	60 (17)		51 (10)		38 (11)	
* * Yes	58 (17)		51 (10)		37 (10)	
Maternal education		<.001		.01		.005
* * None	58 (16)		51 (10)		37 (11)	
					40 (12)	
* * Primary+	64 (17)		54 (11)			
Family possession score (quartile)^c^		<.001		.004		.01
* *1	56 (15)		49 (10)		36 (11)	
					36.5 (10.5)	
* * 2	57 (14.5)		51 (9.5)		39 (12)	
* * 3	62 (14)		52 13)		40 (10)	
* * 4	67.5 (16)		54 (10)			
EPDS score^d^		.03		.07		.62
* * *<*10	58 (17)		51 (11)		38 (12)	
* * >10	62 (14)		52 (10)		38 (9)	
HOME score^e^		.005		.08		.08
* * ** ** *<*27 (mean)	58 (15)		51 (10)		37 (11)	
* * ** **>27	62 (16)		52 (11)		39 (13)	
Child characteristics						
Preterm (<37 weeks’ gestation)		.88		.79		.87
* * No	59 (17)		51 (10)		38 (12)	
* * Yes	58.5 (15)		52 (8)		38.5 (12)	
Low birth weight (<2500 g)		.70		.74		.23
* * No	59 (16)		51 (10)		38 (11)	
* * Yes	58.5 (20)		52 (10)		36 (9)	
Malaria infection^f^		.25		.37		.46
* * No	59 (18.5)		51 (8)		38 (10)	
* * Yes	60 (15)		52 (10)		37 (11)	
Sex		.07		.94		<.001
* * Male	60 (15)		51 (12)		42 (13)	
* * Female	58 (18)		52 (9)		35 (7)	
Blood lead levels^g^		.26		.22		.95
* * ** ** *<*50 μg	61 (18)		51 (10)		37 (10)	
* * ** **>50 μg	59 (15.5)		51 (8)		38 (11)	

Median (interquartile range) assessment scores are shown for each category. Wilcoxon rank-sum and Kruskal-Wallis analyses were used for binary and ordinal variables, respectively. Abbreviations: BMI, body mass index; BOT-2, Bruininks-Oseretsky Test of Motor Proficiency, 2nd edition; EPDS, Edinburgh Postnatal Depression Scale; HOME, Home Observation for Measurement of the Environment; IPTp, intermittent preventive treatments of MiP; KABC-II, Kaufman Assessment Battery for Children, 2nd edition; MiP, malaria in pregnancy; MPI, Mental Processing Index; MQ, mefloquine; NVI, Non-Verbal Index; SP, sulfadoxine-pyrimethamine; STH, soil-transmitted helminth.

^a^Iron deficiency at recruitment, defined as ferritin serum concentration <12 μg/L in the absence of inflammation or a serum ferritin concentration <70 μg/L in the presence of inflammation measured by C-reactive protein.

^b^Women considered to be infected with STHs if the fecal egg count (FEC) was at least 24 eggs per gram for any species.

^c^Family possession score was computed using a checklist of material possessions that families could own and indicated family wealth: radio, television, bicycle, motorbike, car, at least 2 cows, and electricity (quartile 1 = most deprived, quartile 4 = least deprived).

^d^Women with a score of >10 were considered to have symptoms of postpartum depression.

^e^The HOME score is a continuous score based on a combination of interview questions and observations of parent–child interactions in the home and consists of several subscales, with higher scores indicating better-quality parent–child interactions and home learning environments.

^f^Malaria infection at the time of neurocognitive assessment, diagnosed by thick blood smear.

^g^Blood lead levels measured at the time of neurocognitive assessment using inductively coupled plasma mass spectrometry (ICP-MS; Perkin Elmer Sciex Elan DRC II ICP-MS instrument). Cutoff determined by the Centers for Disease Control and Prevention (CDC) intervention threshold for lead poisoning.

### Malaria in Pregnancy and Child Neurocognitive Development

**Figure 2.**
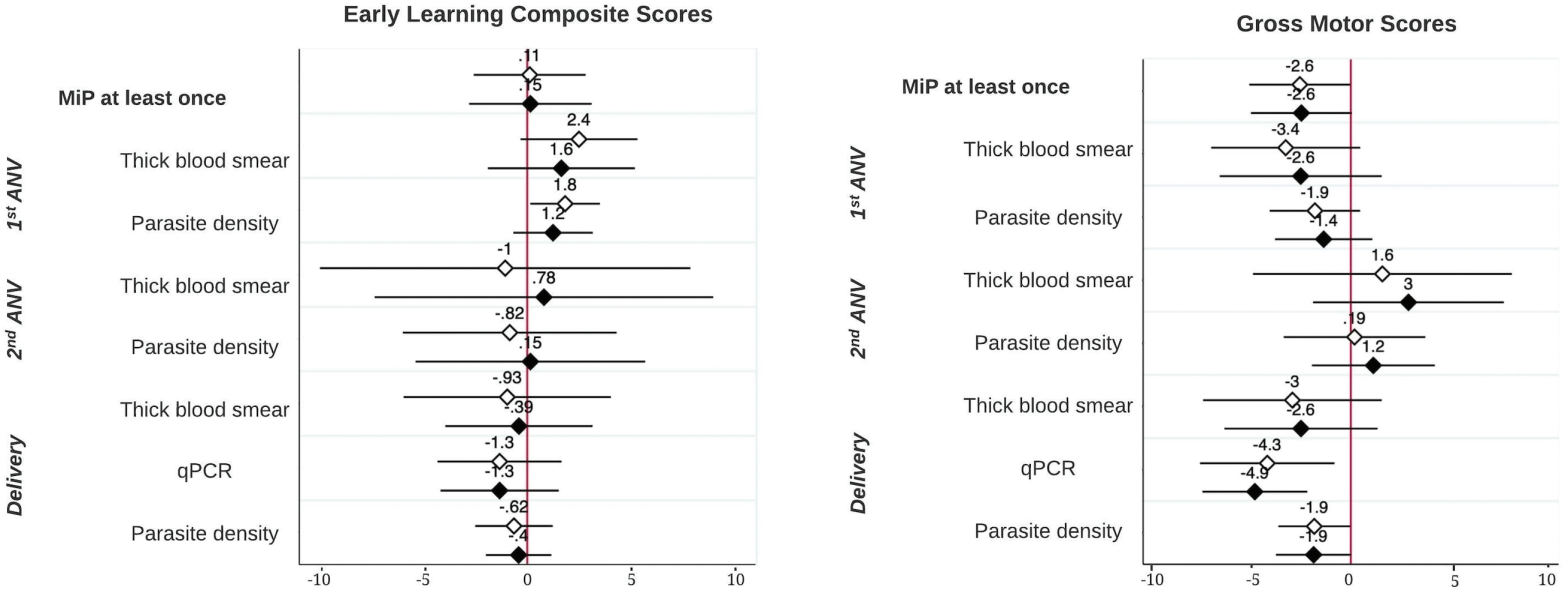
Linear regression B-coefficients and confidence intervals of association between MiP and child neurocognitive development at 1 year of age. White diamond, unadjusted B-coefficients and 95% bootstrapped confidence intervals. Black diamond, adjusted B-coefficients and 95% bootstrapped confidence intervals; controlling for maternal age and education, gravidity, prepregnancy BMI, family possession score, HOME score, and child sex and age at time of neurocognitive assessment. Abbreviations: ANV, antenatal visit; BMI, body mass index; HOME, Home Observation for Measurement of the Environment; MiP, malaria in pregnancy; qPCR, quantitative polymerase chain reaction.

**Figure 3.**
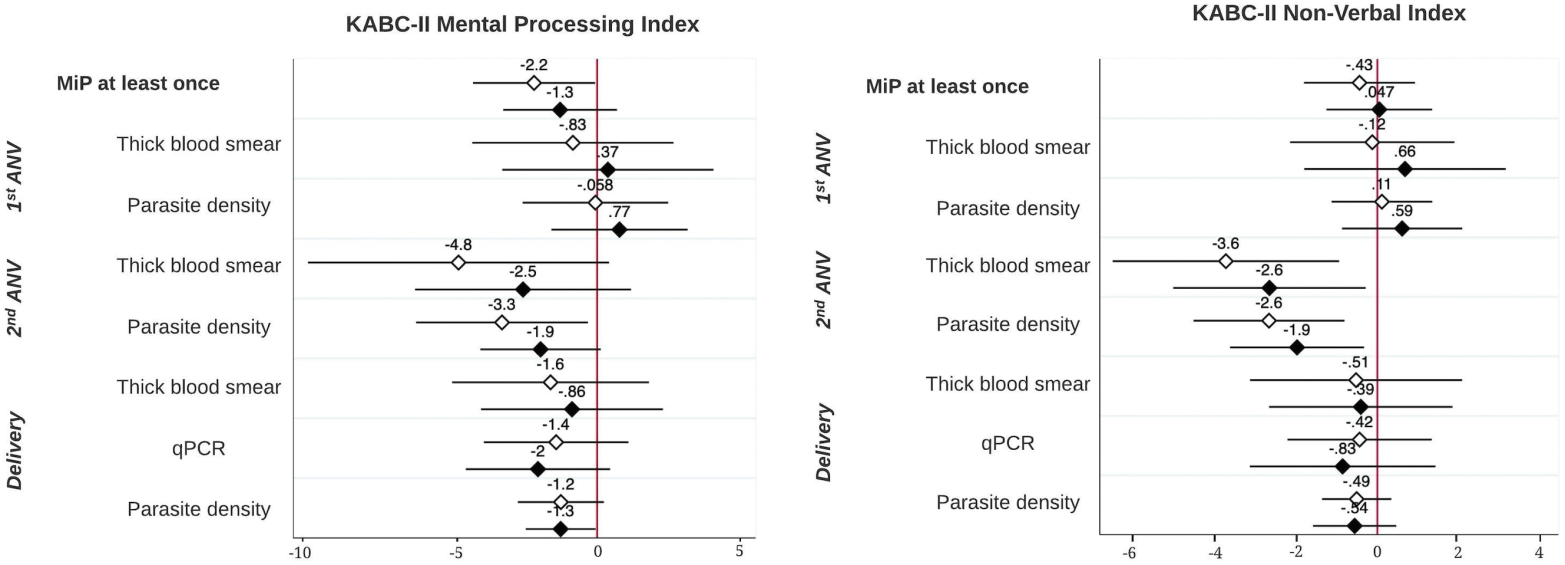
Linear regression B-coefficients and confidence intervals of association between MiP and child neurocognitive development at 6 years of age. White diamond, unadjusted B-coefficients and 95% bootstrapped confidence intervals. Black diamond, adjusted B-coefficients and 95% bootstrapped confidence intervals; controlling for maternal age and education, gravidity, prepregnancy BMI, family possession score, HOME score, and child sex and age at time of neurocognitive assessment. Abbreviations: ANV, antenatal visit; BMI, body mass index; HOME, Home Observation for Measurement of the Environment; KABC-II, Kaufman Assessment Battery for Children, 2nd edition; MiP, malaria in pregnancy; qPCR, quantitative polymerase chain reaction.

Linear regression results between MiP and child neurocognitive and motor development measured by the MSEL at 1 year are shown in Figure 2. While MiP diagnosed at least once during pregnancy did not significantly impair ELC scores in children (0.15; −2.72, 3.01), it did significantly impair gross motor development scores at 1 year before and after adjustment of risk factors (−2.63 [−5.24, −0.02] and −2.55 [−5.15, 0.05], respectively). In particular, malaria diagnosed by qPCR and higher parasite density at delivery were associated with significantly impaired gross motor development scores in children after adjustment (−4.95 [−7.65, −2.24] and −1.92 [−3.86, 0.02], respectively). Malaria in pregnancy diagnosed at any ANV or delivery by thick blood smear was not associated with impaired cognitive or motor development scores in children at 1 year. Figure 3 shows that MiP at least once and high parasite density at the second ANV were associated with impaired overall cognitive development measured by the MPI at 6 years in crude models (−2.17 [−4.27, −0.07] and −3.27 [−6.23, −0.32], respectively); however, these associations were no longer statistically significant after adjustment. High parasite density at delivery was associated with impaired MPI scores in adjusted models (−1.25; −2.46, −0.05). Malaria diagnosed by microscopy and high parasite density at the second ANV were both significantly associated with impaired NVI scores from the KABC-II in adjusted models (−2.57 [95% CI: −4.86, −0.28] and −1.91 [95% CI: −3.51, −0.32], respectively). Late-term malaria infections at the second ANV and delivery showed trends of impairing both MPI and NVI scores at 6 years, although this was statistically nonsignificant. Finally, no measure of MiP was associated with impaired motor composite scores in children at 6 years (Supplementary Figure 1).

### Sensitivity Analyses

Multiple imputation of missing variables (>20%) confirmed results seen in principal analyses (Supplementary Table 1); however, associations between NVI scores and malaria diagnosed at the second ANV were borderline significant. Regression models controlling for preterm birth and low birth weight confirmed principal analyses (Supplementary Table 2). Other factors, such as IPTp treatment, iron deficiency, child blood lead levels, and STH infection, were tested and did not influence the results.

## DISCUSSION

This paper reveals that prenatal exposure to *P. falciparum* malaria has long-term consequences on neurocognitive development in surviving offspring in our cohort. Malaria in pregnancy at least once during pregnancy, placental malaria by qPCR, and high parasite density at delivery are associated with impaired gross motor development scores in children at 1 year. Malaria in pregnancy diagnosed by microscopy and high parasite density at the second ANV negatively impacted NVI scores of the KABC-II, and analyses showed a trend that infections occurring later in pregnancy more negatively impact KABC-II scores than earlier infections. Placental malaria by qPCR was borderline significantly associated with impaired KABC-II scores, and parasite density at delivery had a negative impact on scores after adjustment of risk factors.

The prospective nature of this mother–child cohort and the comprehensive biological and neurocognitive assessments carried out within this population are defining characteristics of this study. This is the first study to our knowledge to investigate the relationship between MiP and its long-term impacts on child neurocognitive development in such a large cohort from SSA. The validity of child development assessments was confirmed and assessments were adapted for use in this population previously [13, 14]. The validity and reliability of MSEL scores at 1 year to predict neurocognitive development at 6 years in this population were also verified in a preceding publication [15]. Sensitivity analyses investigated other potential confounding and intermediate factors, such as the IPTp treatment group, iron deficiency in pregnancy, child blood lead levels, and helminth infection in pregnancy [16], none of which significantly altered the results. Preterm birth and low birth weight are 2 previously hypothesized mechanisms due to their significant impact on neurocognitive development and their associations with submicroscopic malarial infections during pregnancy [17–19]. However, results excluding preterm and low-birth-weight infants were unchanged; our results therefore do not suggest a mechanism through these hypothesized factors. Multiple imputation confirmed our results, ruling out potential bias from missingness. Finally, there were no differences in terms of MiP exposure in women who were included and excluded from analyses; therefore, attrition bias is unlikely.

There are several limitations to our analyses, including the limited sample size in analyses for placental malaria and the multitude of tests conducted in this population, increasing the likelihood that results could be found by chance. The use of the Ballard score for gestational age at birth is also a limitation as this could have introduced measurement error when defining preterm birth. Pregnant women and children were followed extensively and treated promptly in the case of malaria infection, therefore potentially underestimating associations between MiP and child neurocognitive development. Due to the close follow-up, our study was also unable to investigate the impacts of severe malaria during pregnancy and childhood, known to have detrimental neurological consequences on survivors [20]. However, sensitivity analyses also tested the impact of repeated malaria episodes in children between 1 and 6 years of age on the main associations and found no changes. Last, pregnant women were not recruited until the second trimester of pregnancy and so this study was unable to expand upon the limited existing literature on the impact of infections in the first trimester on poor birth outcomes [21] and neurocognitive development in offspring.

Research on the timing of maternal infections during pregnancy and its impact on fetal health remains equivocal [22, 23]. Our analyses show a trend between late-term infection and lower neurocognitive scores in children at 1 and 6 years. The World Health Organization recently revised their standard recommendation for IPTp in high-transmission areas from 2 doses during pregnancy to 3 doses to help improve birth outcomes [24]. Therefore, the higher proportion of women diagnosed with malaria at delivery could reflect chronic infections due to women not receiving tertiary IPTp doses between the second ANV and delivery in the clinical trial.

Previous studies have shown the effect of maternal infection during pregnancy on fetal neurodevelopmental abnormalities and risk of psychiatric disorders [25–27], with authors hypothesizing that maternal immune activation in response to infection can have severe consequences on neurological development of the fetus. However, these studies either overlooked the effect of malaria entirely or malaria was assessed as a composite of overall maternal infection during pregnancy. A laboratory-based study provided evidence of a causal link between experimental malaria infection in pregnancy and impaired neurocognitive development in the fetus [6, 7]. Similar to previously mentioned hypotheses, their research suggests that complement factors of the innate maternal immune response to infection, in particular C5a, initiate proinflammatory and antiangiogenic pathways that alter neurovascular development of the fetus in the placenta. Our analyses did not investigate the mechanistic role of maternal inflammation; however, future cohort studies should do so in order to add to the limited existing research.

Placental malaria and parasite density at delivery, diagnosed by qPCR, were found to be particularly detrimental to development scores. One explanation for this could be that submicroscopic placental malaria infections may be particularly deleterious to fetus growth and development [19, 28, 29]. While qPCR technology is able to identify submicroscopic malarial infections undetectable by microscopy, laboratory resources are not readily available in low-income countries like Benin. Therefore, women with submicroscopic infections in our population were not immediately diagnosed and treated at the time of delivery and these infections could reflect more chronic infections that are not easily diagnosed by microscopy methods and treated rapidly.

This research provides evidence of neurocognitive consequences on fetuses exposed to peripheral and placental malaria during pregnancy and expands on existing epidemiological knowledge of malaria in pregnancy in endemic areas. Submicroscopic, asymptomatic malarial infections pose a high risk to pregnant women in malaria-endemic countries like Benin, and sufficient IPTp during antenatal care, as well as in early childhood [30], is vital to address their risks on fetal development. This work reinforces the need for routine antenatal care visits to adequately diagnose and treat *P. falciparum* infections in pregnant women. Further studies in large cohorts should be conducted to confirm our analyses and expand upon hypothesized mechanisms. Further research on clinical representations and diagnosis of neurocognitive outcomes and disability is also greatly needed in African cohorts; this population is being followed for 11 years postpartum in order to better interpret results from neurocognitive assessments and understand their norms and clinical representations.

## Supplementary Data

Supplementary materials are available at *Clinical Infectious Diseases* online. Consisting of data provided by the authors to benefit the reader, the posted materials are not copyedited and are the sole responsibility of the authors, so questions or comments should be addressed to the corresponding author.

ciab569_suppl_Supplementary_Figure_S1

ciab569_suppl_Supplementary_Table_S1

ciab569_suppl_Supplementary_Table_S2

## Notes

***Acknowledgments.*** The authors thank the on-site researchers and public health nurses in Allada, Benin, for their collaboration. They thank Nicaise Tuikue Ndam for their contribution in qPCR analyses.

***Financial support.*** This work was supported by the Fondation de France (grant number 2015 00060746 to F. B.-L.), the Eunice Kennedy Shriver National Institute of Child Health and Human Development (grant number NIH/NICHD- R21-HD060524 to F. B.-L.), the European and Developing Countries Clinical Trials Partnership (grant number EDCTP- IP.07.31080.002 to M. C.], and the Bill and Melinda Gates Foundation (to M. C.).

***Potential conflicts of interest.*** The authors: No reported conflicts of interest. All authors have submitted the ICMJE Form for Disclosure of Potential Conflicts of Interest.
