# Supplementary material for: The Effects of Malaria in Pregnancy on Neurocognitive Development in Children at 1 and 6 Years of Age in Benin: A Prospective Mother–Child Cohort
Source: Clin Infect Dis. 2021 Jul 23;74(5):766–75. doi: 10.1093/cid/ciab569 (PMC8906760; doi:10.1093/cid/ciab569)
Supplement: ciab569_suppl_Supplementary_Figure_S1 [file ciab569_suppl_supplementary_figure_s1.pdf]

Supplemental Figure 1. Linear regression beta coefficients and confidence intervals of association between MiP and child gross motor development at one year of age.

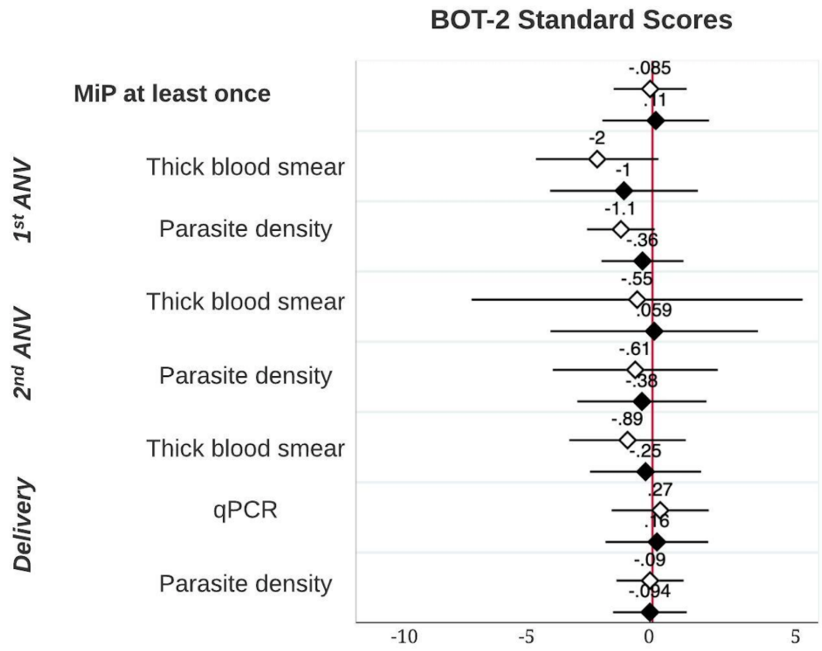

-◇-- Unadjusted beta-coefficients and 95% bootstrapped confidence intervals.

-◆-- Adjusted beta-coefficients and 95% bootstrapped confidence intervals; controlling for maternal age and education, gravidity, pre-pregnancy BMI, family possession score, HOME score, and child sex and age at time of neurocognitive assessment.
