# Supplementary material for: The Effects of Malaria in Pregnancy on Neurocognitive Development in Children at 1 and 6 Years of Age in Benin: A Prospective Mother–Child Cohort
Source: Clin Infect Dis. 2021 Jul 23;74(5):766–75. doi: 10.1093/cid/ciab569 (PMC8906760; doi:10.1093/cid/ciab569)
Supplement: ciab569_suppl_Supplementary_Table_S1 [file ciab569_suppl_supplementary_table_s1.pdf]

Supplemental Table 1. Linear regression with multiple imputation of missing variables (N=493).

	MSEL Early Learning Composite (ELC) Score		MSEL Gross Motor Score	
	Crude	Adjusted[1]	Crude	Adjusted[1]
**Thick blood smear at 1st ANV**	2.35 (-1.26, 5.95)	1.81 (-1.89, 5.51)	-3.41 (-7.10, 0.28)	-2.64 (-6.49, 1.21)
**Parasite density at 1st ANV**	1.69 (-0.58, 3.96)	1.25 (-1.09, 3.59)	-1.95 (-4.25, 0.34)	-1.49 (-3.89, 0.92)
**Thick blood smear at 2nd ANV**	-0.97 (-7.61, 5.67)	0.31 (-6.17, 6.79)	1.83 (-4.82, 8.49)	2.49 (-4.14, 9.11)
**Parasite density at 2nd ANV**	-0.80 (-5.00, 3.39)	-0.14 (-4.21, 3.94)	0.21 (-3.99, 4.42)	0.77 (-3.39, 4.94)
**Thick blood smear at delivery**	-1.03 (-5.17, 3.11)	-0.38 (-4.49, 3.74)	-2.93 (-7.12, 1.26)	-2.70 (-6.93, 1.53)
**qPCR of placenta at delivery**	-0.97 (-3.89, 1.94)	-0.94 (-3.77, 1.88)	-3.59 (-6.56, -0.63) *	-4.07 (-6.98, -1.16) **
**Parasite density at delivery**	-0.58 (-2.39, 1.22)	-0.59 (2.34, 1.16)	-1.82 (-3.63, -0.01) *	-1.99 (-3.79, -0.21) *
**MiP at least once in pregnancy**	0.07 (-2.54, 2.68)	0.17 (-2.43, 2.76)	-2.66 (-5.24, -0.08) *	-2.51 (-5.13, 0.10)

	KABC-II Mental Processing Index		KABC-II Non-Verbal Index	
	Crude	Adjusted[1]	Crude	Adjusted[1]
**Thick blood smear at 1st ANV**	-0.90 (-3.99, 2.18)	0.24 (-2.78, 3.26)	-0.11 (-2.11, 1.89)	0.52 (-1.50, 2.55)
**Parasite density at 1st ANV**	-0.10 (-2.04, 1.84)	0.61 (-1.31, 2.53)	0.11 (-1.15, 1.37)	0.49 (-0.80, 1.77)
**Thick blood smear at 2nd ANV**	-4.30 (-10.18, 1.57)	-3.08 (-8.61, 2.45)	-3.73 (-7.70, 0.25)	-2.99 (-6.85, 0.86)
**Parasite density at 2nd ANV**	-2.92 (-6.68, 0.83)	-2.21 (-5.75, 1.32)	-2.61 (-5.18, -0.03) *	-2.15 (-4.65, 0.34)
**Thick blood smear at delivery**	-1.77 (-5.37, 1.83)	-1.23 (-4.66, 2.20)	-0.67 (-3.01, 1.67)	-0.59 (-2.90, 1.72)
**qPCR of placenta at delivery**	-1.70 (-4.21, 0.81)	-1.83 (-4.19, 0.52)	-0.55 (-2.17, 1.06)	-0.66 (-2.24, 0.91)
**Parasite density at delivery**	-1.29 (-2.84, 0.26)	-1.31 (-2.77, 0.14)	-0.48 (-1.49, 0.52)	-0.54 (-1.51, 0.44)
**MiP at least once in pregnancy**	-2.24 (-4.41, -0.06) *	-1.40 (-3.48, 0.68)	-0.49 (-1.91, 0.93)	-0.08 (-1.48, 1.32)

[1]Adjusted for maternal age, education, pre-pregnancy BMI, family possession score, gravidity, child sex and age at time of assessment
*p-value≤0.05
** p-value≤0.01
