# Supplementary material for: The Effects of Malaria in Pregnancy on Neurocognitive Development in Children at 1 and 6 Years of Age in Benin: A Prospective Mother–Child Cohort
Source: Clin Infect Dis. 2021 Jul 23;74(5):766–75. doi: 10.1093/cid/ciab569 (PMC8906760; doi:10.1093/cid/ciab569)
Supplement: ciab569_suppl_Supplementary_Table_S2 [file ciab569_suppl_supplementary_table_s2.pdf]

Supplemental Table 2. Linear regression controlling for risk factors and intermediate factors (pre-term birth and low birth weight).

	MSEL Early Learning Composite (ELC) Score		MSEL Gross Motor Score	
	Crude	Adjusted[1]	Crude	Adjusted[1]
**Thick blood smear at 1st ANV**	2.42 (-0.31, 5.15)	1.06 (-1.92, 4.05)	-3.36 (-7.20, 0.48)	-2.75 (-6.28, 0.78)
**Parasite density at 1st ANV**	1.77 (0.14, 3.39) *	0.90 (-0.84, 2.65)	-1.85 (-4.18, 0.48)	-1.54 (-3.47, 0.38)
**Thick blood smear at 2nd ANV**	-1.03 (-9.69, 7.62)	0.54 (-7.02, 8.11)	1.63 (-5.05, 8.30)	2.32 (-3.52, 8.17)
**Parasite density at 2nd ANV**	-0.82 (-5.81, 4.18)	-0.05 (-5.32, 5.23)	0.19 (-3.46, 3.84)	0.91 (-2.65, 4.48)
**Thick blood smear at delivery**	-0.93 (-5.78, 3.91)	-0.83 (-4.45, 2.80)	-3.01 (-7.62, 1.60)	-2.76 (-6.52, 1.01)
**qPCR of placenta at delivery**	-1.29 (-4.19, 1.60)	-1.60 (-4.45, 1.25)	-4.31 (-7.78, -0.84) *	-5.07 (-8.45, -1.68) **
**Parasite density at delivery**	-0.62 (-2.43, 1.19)	-0.82 (-2.48, 0.83)	-1.89 (-3.73, -0.04) *	-2.27 (-4.09, -0.45) **
**MiP at least once in pregnancy**	0.11 (-2.50, 2.73)	-0.68 (-3.10, 1.74)	-2.63 (-5.24, -0.02) *	-3.06 (-5.72, -0.40) *

	KABC-II Mental Processing Index		KABC-II Non-Verbal Index	
	Crude	Adjusted[1]	Crude	Adjusted[1]
**Thick blood smear at 1st ANV**	-0.83 (-4.29, 2.63)	0.78 (-3.12, 4.68)	-0.12 (-2.08, 1.84)	0.72 (-1.42, 2.86)
**Parasite density at 1st ANV**	-0.06 (-2.56, 2.44)	1.09 (-1.49, 3.68)	0.11 (-1.09, 1.31)	0.71 (-0.61, 2.02)
**Thick blood smear at 2nd ANV**	-4.77 (-9.94, 0.41)	-3.24 (-7.70, 1.21)	-3.61 (-6.30, -0.91) **	-2.49 (-4.98, 0.01) *
**Parasite density at 2nd ANV**	-3.27 (-6.23, -0.32) *	-2.23 (-4.46, -0.01) *	-2.58 (-4.38, -0.78) **	-1.83 (-3.54, -0.12) *
**Thick blood smear at delivery**	-1.60 (-4.98, 1.78)	-0.94 (-4.19, 2.30)	-0.51 (-3.03, 2.02)	-0.18 (-3.00, 2.65)
**qPCR of placenta at delivery**	-1.41 (-3.89, 1.08)	-2.02 (-4.80, 0.77)	-0.42 (-2.14, 1.29)	-0.76 (-2.74, 1.23)
**Parasite density at delivery**	-1.25 (-2.73, 0.23)	-1.30 (-2.71, 0.12)	-0.49 (-1.32, 0.33)	-0.45 (-1.49, 0.59)
**MiP at least once in pregnancy**	-2.17 (-4.27, -0.07) *	-1.20 (-3.78, 1.39)	-0.43 (-1.75, 0.90)	0.19 (-1.48, 1.86)

[1]Adjusted for maternal age, education, pre-pregnancy BMI, family possession score, gravidity, child sex and age at time of assessment + pre-term birth and low birth weight

*p-value≤0.05

** p-value≤0.01
